# Coexistence of PMQR and ESBL genes among clinical *Escherichia coli* isolates from community-acquired UTI in Mexicali, on the US-Mexico border

**DOI:** 10.1016/j.bjid.2025.104554

**Published:** 2025-05-23

**Authors:** Dolores A. Marquez-Salazar, Ricardo Delgadillo-Valles, Gerson N. Hernandez-Acevedo, Edwin Barrios-Villa, Raquel Muñiz-Salazar, Gilberto Lopez-Valencia, Paulina Haro, Enrique Trasviña-Muñoz, Rafael Martinez-Miranda, Jonathan Arauz-Cabrera

**Affiliations:** a Universidad Autónoma de Baja California, Facultad de Medicina Mexicali, Departamento de Farmacología, Humberto Torres Sanginés SN, Centro Cívico, Mexicali, Baja California, Mexico; b Universidad Autónoma de Baja California, Facultad de Medicina Mexicali, Departamento de Microbiología y Parasitología Clínica, Humberto Torres Sanginés SN, Centro Cívico, Mexicali, Baja California, Mexico; c Universidad de Sonora, Campus Caborca, Departamento de Ciencias Químico Biológicas y Agropecuarias, Laboratorio de Biología Molecular y Genómica Sonora, Mexico; d Universidad Autónoma de Baja California, Escuela de Ciencias de la Salud, Unidad Valle Dorado, Campus Ensenada, Laboratorio de Epidemiología y Ecología Molecular, Baja California, Mexico; e Universidad Autónoma de Baja California, Instituto de Investigaciones en Ciencias Veterinarias, Baja California, Mexico; f Hospital Almater, Departamento de Microbiología, Mexicali, Baja California, Mexico

**Keywords:** *Escherichia coli*, Resistotype, Community-acquired infection, ESBL, PMQR

## Abstract

*Escherichia coli* is an opportunistic pathogen and a leading cause of Community-Acquired Urinary Tract Infections (CA-UTIs). *E. coli* can harbor multiple genetic resistant determinants, such as Extended Spectrum Beta-lactamases (ESBL) and Plasmid-Mediated Quinolone Resistance (PMQR) genes, complicating the empirical treatment of UTIs with β-lactams and quinolones. The aim of his study was to characterize ESBL and PMQR genes among *E. coli* isolates from CA-UTI in Mexicali, Mexico Isolates were collected from January to December 2023. Identification was performed by MALDI-TOF, and ESBL-producing determination and antimicrobial susceptibility by Vitek 2. Detection of ESBL and PMQR, and phylotyping were performed by PCR. Genetic diversity was determined by Enterobacterial Repetitive Intergenic Consensus Polymerase Chain Reaction (ERIC-PCR). Eighty-nine *E. coli* isolates were collected from CA-UTIs. All exhibited resistance to ciprofloxacin, ceftriaxone, and cefotaxime, while being susceptible to carbapenems and ceftazidime/avibactam. All isolates tested positive for an ESBL gene, with *bla_CTX−__M_* (98.9 %) being the most prevalent. Five isolates tested negative for PMQR genes; the remaining showed *aac(6′)-lb-cr* present in 85.3 %. Coexistence between ESBL and PMQR genes was noted in 95.5 %. Most prevalent plylogenetic group was group B2 (74.2 %). This study provides valuable regional data, highlighting a public health problem due to the high prevalence of ESBL and PMQR genes in *E. coli* responsible for CA-UTI, which are linked to multidrug resistance. Genetic diversity was found, suggesting multiple sources of resistant strains in the community. These findings underline the need for surveillance and control to limit the spread of resistant *E. coli*, locally and globally.

**Data summary:**

The authors confirm all supporting data, code, and protocols have been provided within the article or through supplementary data files.

## Introduction

*E. coli* is a Gram-negative bacillus resident of gut microbiota and serves as an opportunistic pathogen.^1^ It is a prominent cause of community- and hospital-acquired infections.^2^ *E. coli* has been implicated in Urinary Tract Infections (UTIs), which are the most common infections.^3^ However, it has also exhibited a concerning trend of increasing antibiotic resistance globally, primarily attributed to its ability to harbor multiple genetic resistant determinants such as Extended Spectrum Beta-lactamases (ESBLs)^2^ and Plasmid-Mediated Quinolone Resistance (PMQR) genes, mostly through Mobile Genetic Elements (MGE).^3^

Resistance to third-generation cephalosporins is rising, leading to increased attention on ESBL enzymes, which can hydrolyze the β-lactam ring in a wide range of β-lactam antimicrobials. This has heightened reliance on last-resort antimicrobial drugs, such as carbapenems. The most prevalent ESBLs are encoded by *bla_TEM_, bla_SHV_* and *bla_CTX−__M_*, with the frequency of *bla_CTX−__M_* being the highest in ESBL-producing *E. coli*.^4^ CTX-M variants can be classified into five major groups based on their amino acid sequence similarity: CTX-M Group-1, −2, −8, −9, and 25/26. Among these, CTX-M Group 1 includes CTX-M15, a widely distributed variant and associated with pandemic clones.^4^

The distribution of ESBL genes may vary worldwide; however, reports from Mexico indicate that Enterobacterales, including *E. coli,* can harbor *bla_TEM_, bla_SHV_* and *bla_CTX−__M_*,^3^^,^^5^^,^^6^ as well as PMQR genes such as *qnr, qepA,* and *aac(6′)-Ib-cr*.^3^^,^^5^ The presence of these genes in a strain represents a challenge for treatment, as they confer resistance to β-lactams and quinolones, respectively, which are antimicrobial agents commonly used as empirical treatment for UTI.^7^

To describe the genetic characteristics of populations, such as associated pathotypes and serotypes, classification schemes, including phylotyping, have been established.^8^ Additionally, techniques such as Enterobacterial Repetitive Intergenic Consensus Polymerase Chain Reaction (ERIC-PCR), have been developed for genetic screening and fingerprinting.^9^

Mexicali is a community shared between Southern California and northwest Mexico. It serves as a medical tourism destination in northern Mexico, attracting patients from both sides of the border. Although previous studies have assessed the prevalence of ESBL and PMQR genes within healthcare facilities in Mexico,^5^^,^^10^, ^11^, ^12^ there is limited research focused on community settings.^3^^,^^10^^,^^12^^,^^13^

Given the unique characteristics of our community setting, this study aimed to determine the resistance patterns to antimicrobials and the prevalence of ESBL and PMQR genes in *E. coli* Community-Acquired Urinary Tract Infections (CA-UTIs) isolates obtained from patients in Mexicali, Mexico.

## Methods

### Setting and sample collection

The present study was conducted in Mexicali (32°37′40.1′' N 115°27′16.1′' W), a border city between northwestern Mexico and southern California, United States. *E. coli* strains were collected from community patients between January and December 2023. The strains were isolated from urine samples with a colony count exceeding 100 000 CFU.

### Species identification and antimicrobial susceptibility testing

*E. coli* strains were identified using mass spectrometry MALDI-TOF (Bruker Daltonics, Bremen, Germany); ESBL production and antimicrobial susceptibility tests were performed by Vitek 2 system (BioMerieux, Marcy- l’Ètoile, France) according to the Clinical and Laboratory Standards Institute guidelines.^14^ The strains were tested for susceptibility towards the following agents: Ampicillin (AM), Amikacin (AN), Ceftazidime (CAZ), Cephalothin (CEF), Ciprofloxacin (CIP), Ceftriaxone (CRO), Cefotaxime (CTX), Cefuroxime (CXM), Doripenem (DOR), Ertapenem (ETP), Cefepime (FEP), Fosfomycin (FOS), Nitrofurantoin (FT), Gentamicin (GM), Imipenem (IPM), Levofloxacin (LEV), Meropenem (MEM), Ampicillin/Sulbactam (SAM), Trimethoprim/Sulfamethoxazole (STX), ceftazidime/avibactam, with AST-N71, AST-N72 and AST-X05 reagents. Subsequently, isolates were stored in Trypticase Soy Broth (TSB) with 15 % glycerol at −70 °C.

### Molecular characterization of the isolates, ESBL and PMQR genes detection

Isolates exhibiting ESBL production and Ciprofloxacin resistance were selected for molecular analysis. Strains were incubated in Trypticase soy agar for 18–24-hours at 36 °C. One colony from each strain was grown overnight in 3 mL of TSB, then vortex homogenized, and 1.5 mL was transferred to a new tube. DNA extraction was performed using the Plasmid DNA extraction kit (GeneNano, Rochester, New York, USA) with a modified protocol to obtain both chromosomal and plasmid DNA. DNA concentration and purity were assessed using a NanoDrop-1000 Spectrophotometer (Thermo Fisher Scientific, Waltham Massachusetts, USA). All DNA extractions had an A260/280 ratio above 1.8, and concentrations were diluted ranging from 100 to 200 ng/µL. Clinical isolates were confirmed by PCR using primers for the *ybbW* gene, which is highly specific for *E. coli* and encodes an allantoin receptor.^15^^,^^16^

Detection of ESBL genes (*bla_TEM_, bla_SHV_, bla_CTX−__M_*_group-1_^29^ *bla_CTX−__M_*_group-2_*, bla_CTX−__M_*_group-8_*, bla_CTX−__M_*_group-9_*,* and *bla_CTX−__M-151_*)*,* and PMQR genes *(qepA, qnrB, oqxA, oqxB,* and *aac(6′)-lb-cr)*^30^ was performed by PCR, according the conditions outlined in Supplementary Table 1. Phylotyping was carried out using a previously established method based on the detection of *arpA, chuA, yjaA*, genes, and TspE4.C2 chromosomic fragment by using a quadruplex PCR.^17^

ERIC-PCR was performed to determine clonal diversity among the isolates as previously described.^9^ *E. coli* ATCC25922 was used as control strain. Agarose Gel Electrophoresis (AGE) was performed on 2 % agarose gels, and images were analyzed using GelJ v2^18^ with the Dice method. A dendrogram was constructed using the Unweighted-Pair Group Method with Arithmetic Means (UPGMA) and visualized with iTOL.^19^

### Statistical analysis

The prevalence of ESBL and PMQR genes in ESBL-producing *E. coli* strains was determined by calculating the ratio of the number of positive cases for each resistance gene to the total number of samples analyzed, using Statistix 9 software.

## Results

Eighty-nine *E. coli* isolates were collected from CA-UTI, 66.3 % from female patients and 33.7 % from males. Most isolates were recovered from patients over 40 years-old (Table 1).

**Table 1:** Distribution of *E. coli* CA-UTI isolates by sex and age group.

Age	Female	Male
**< 4**	3	0
**5 ‒ 14**	0	0
**15 ‒ 24**	0	0
**25 ‒ 34**	3	1
**35 ‒ 44**	9	0
**45 ‒ 54**	9	6
**55 ‒64**	11	9
**65 ‒ 74**	9	6
**75 ‒ 84**	8	6
**85 ‒ 94**	5	2
**> 95**	2	0

Age is expressed as years (*n* = 89).

### Antimicrobial resistance profile

All strains exhibited resistance towards ampicillin, ceftazidime, cephalothin, ciprofloxacin, ceftriaxone, cefotaxime, levofloxacin, and ampicillin/sulbactam. In contrast, all strains were susceptible to the tested carbapenems (doripenem, ertapenem, imipenem, meropenem) and ceftazidime/avibactam (Table 2).

**Table 2:** Antimicrobial resistance profile of clinical *E. coli* isolates recovered from community patients.

Drug class	Antimicrobial	S	I	R
Aminoglycoside	Amikacin	86/89 (96.6 %)	1/89 (1.1 %)	2/89 (2.2 %)
	Gentamicin	37/88 (42.0 %)	0/88 (0.0 %)	51/88 (57.9 %)
β-lactam and β-lactamase inhibitor	Ceftazidime / Avibactam	79/79 (100 %)	0/79 (0.0 %)	0/79 (0.0 %)
	Ampicillin / Sulbactam	0/89 (0.0 %)	0/89 (0.0 %)	89/89 (100 %)
Carbapenem	Doripenem	3/3 (100 %)	0/3 (0.0 %)	0/3 (0.0 %)
	Ertapenem	88/88 (100 %)	0/88 (0.0 %)	0/88 (0.0 %)
	Imipenem	5/5 (100 %)	0/5 (0.0 %)	0/5 (0.0 %)
	Meropenem	89/89 (100 %)	0/89 (0.0 %)	0/89 (0.0 %)
Cephalosporin	Cefepime	1/89 (1.12 %)	0/89 (0.0 %)	88/89 (98.8 %)
	Cefotaxime	0/83 (0.0 %)	0/83 (0.0 %)	83/83 (100 %)
	Ceftazidime	0/87 (0.0 %)	0/87 (0.0 %)	87/87 (100 %)
	Ceftriaxone	0/89 (0.0 %)	0/89 (0.0 %)	89/89 (100 %)
	Cefuroxime	1/89 (1.12 %)	0/89 (0.0 %)	88/89 (98.8 %)
	Cephalothin	0/46 (0.0 %)	0/46 (0.0 %)	46/46 (100 %)
Nitrofuran	Nitrofurantoin	81/89 (91.0 %)	7/89 (7.8 %)	1/89 (1.1 %)
Penicillin	Ampicillin	0/33 (0.0 %)	0/33 (0.0 %)	33/33 (100 %)
Phosphonic acid derivate	Fosfomycin	83/88 (94.3 %)	1/88 (1.1 %)	4/88 (4.5 %)
Quinolone	Ciprofloxacin	0/89 (0.0 %)	0/89 (0.0 %)	89/89 (100 %)
	Levofloxacin	0/88 (0.0 %)	0/88 (0.0 %)	88/88 (100 %)
Sulfonamides	Trimethoprim/ Sulfamethoxazole	35/89 (39.3 %)	0/89 (0.0 %)	54/89 (60.6 %)

S, Susceptible; I, Intermediate; R, Resistant.

Data is shown as the number of incidences over the total of isolates tested for each antimicrobial; on the second row the corresponding percentage is written.

Twenty-nine distinct antimicrobial resistance profiles were identified. These profiles share resistance to penicillins combined with a β-lactamase inhibitor, cephalosporins, and quinolones.

### ESBL and PMQR genes

**Fig. 1:**
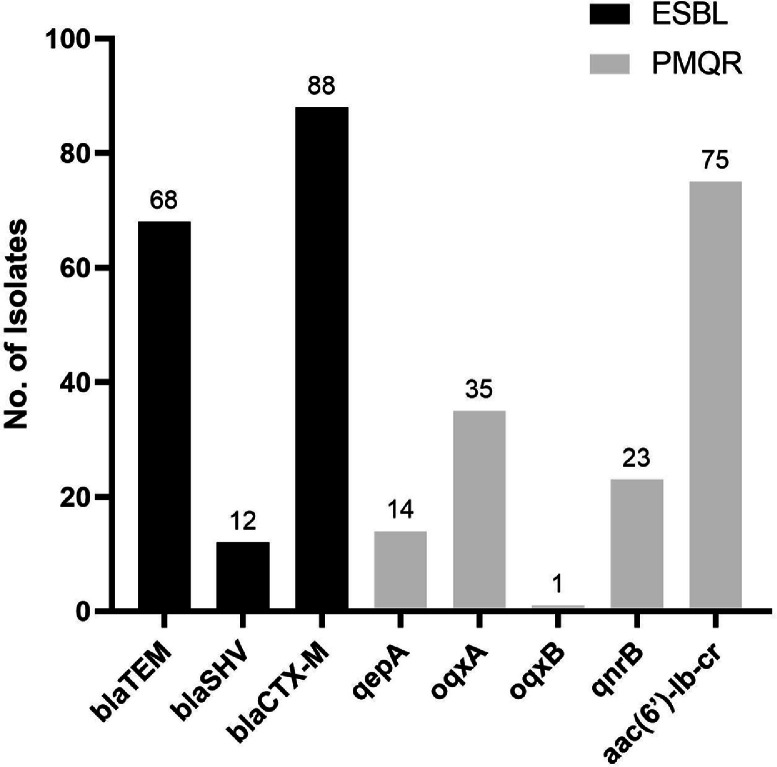
Frequency of antimicrobial resistance genetic determinants among the isolated strains. ESBL (*bla_TEM_, bla_SHV_, bla_CTX−__M_*) and PMQR genes (*oqxA, oqxB, qnrB, qepA, aac(6′)-lb-cr*) *n* = 89.

All strains tested positive for at least one of the three ESBL types analyzed. The most frequently detected gene was *bla_CTX−__M_,* with 88 out of 89 isolated testing positive. This was followed by *bla_TEM_* (76.4 %), and lastly, *bla_SHV_* (13.4 %) (Fig. 1). Among CTX-M groups, Group 2 was the most prevalent (97.8 %), followed by group-1 (76.4 %), Group 8, and Group 9 (both with 18.9 %). The coexistence of ESBL genes and CTX-M groups was observed in several strains. The co-occurrence of CTX-M groups was observed in 86 of the isolates (Table 3) Regarding PMQR genes, the distribution was as follows: *aac(6′)-lb-cr* (83.0 %), *oqxA* (40.6 %), *qnrB* (29.2 %), *qepA* (16.0 %) and *oqxB* (1.9 %). Notably, five isolates tested negative for PMQR genes. Additionally, coexistence of PMQR genes was observed in 50 % of the positive isolates. PMQR and ESBL genes co-occurrence was also noted, resulting in 30 genotypes (Table 4). There was no significant difference (*p* ≥ 0.05) in the prevalence of these genes among isolates based on the patients’ sex.

**Table 3:** Distribution and coexistence of CTX-M groups among isolates (*n* = 88).

CTX-M groups				N° of isolates
Group-1	Group-2	Group-8	Group-9	
+	+	–	–	66
–	+	+	–	3
–	+	+	+	15
+	+	–	+	2
–	+	–	–	1
–	–	–	+	1

+, Positive, -, Negative.

**Table 4:** Coexistence of ESBL and PMQR genes in *E. coli* CA-UTI isolates (*n* = 88).

N° of genes	PMQR gene				ESBL gene			N° of isolates
	*acc(6′)-Ib-cr*	*qnrB*	*oqxAB*	*qepA*	TEM	SHV	CTX-M	
2 genes	+	–	–	–	–	–	+	8
	–	–	–	–	+	–	+	3
	–	–	+	–	–	–	+	1
	–	+	–	–	–	–	+	1
3 genes	+	–	–	–	+	–	+	25
	+	–	+	–	–	–	+	2
	+	+	–	–	–	–	+	2
	–	+	–	+	–	–	+	1
	+	–	–	–	–	+	+	1
	–	–	+	–	+	–	+	2
4 genes	+	–	+	–	+	–	+	7
	+	+	–	–	+	–	+	3
	–	–	+	+	+	–	+	2
	+	–	–	–	+	+	+	4
	+	–	+	+	–	–	+	2
	+	+	+	–	–	–	+	1
	+	+	–	+	–	–	+	1
	+	+	+	–	–	–	+	1
	+	+	–	+	–	–	+	1
	–	–	+	+	+	–	+	1
	–	+	+	–	+	–	+	1
	+	–	–	+	+	–	+	1
	+	+	–	–	+	–	+	1
5 genes	+	+	+	–	+	–	+	4
	+	–	+	+	+	–	+	4
	+	–	+	–	+	+	+	1
	+	–	+	–	+	+	+	1
	+	+	–	–	+	+	+	1
6 genes	+	+	+	–	+	+	+	4
	+	+	+	+	+	–	+	1

+, Positive, -, Negative.

### Phylotyping and genetic diversity

Phylotyping revealed that 68.9 % of the isolates belong to Group B2, 16.9 % to Group A, 5.6 % to Group F, and 3.7 % to Group B1 (66, 15, 5, and 2 isolates, respectively). Only 1 (1.1 %) isolate was identified as a member of Group E and there were zero cases for Group C.

**Fig. 2:**
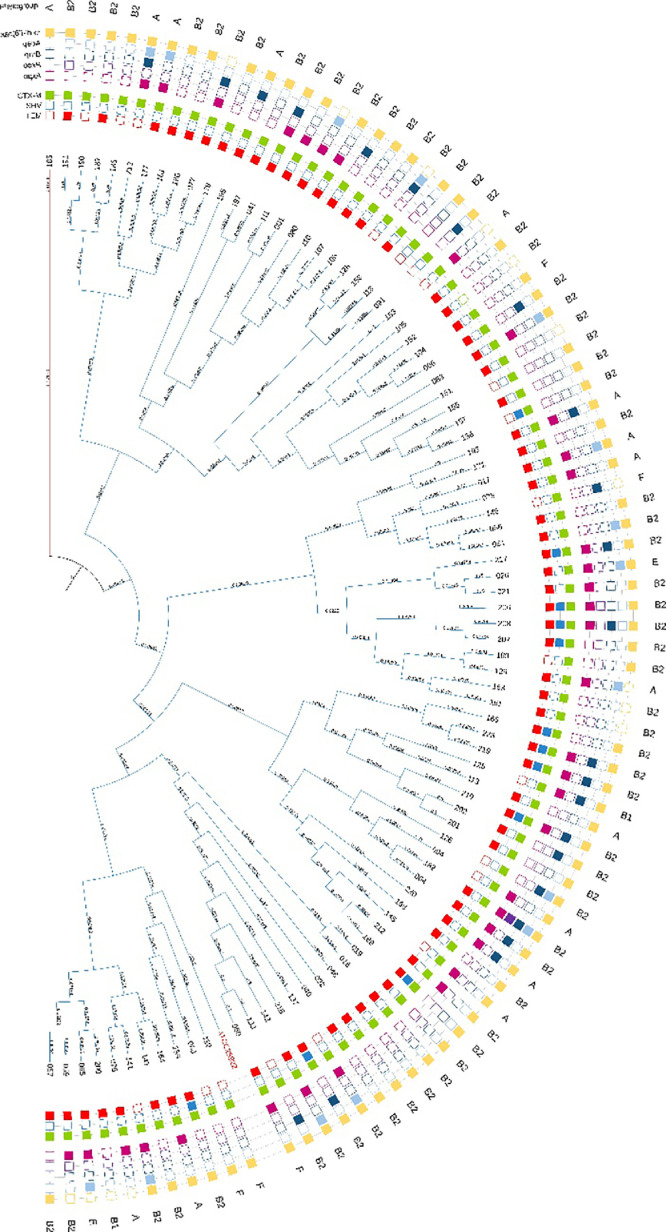
Dendrogram of *E. coli* isolates, based on ERIC-PCR patterns. Genetic distance is indicated on every branch. Isolate identification is located at the end of each branch, followed by ESBL (*bla_TEM_, bla_SHV_, bla_CTX−__M_*), PMQR genes (*oqxA, oqxB, qnrB, qepA, aac(6′)-lb-cr*) and phylogenetic group. Filled cubes represent the presence of the corresponding gene.

ERIC-PCR analysis revealed two clades separated at the origin (Fig. 2). The largest clade was further subdivided into two major subclades, containing 88 of the 89 isolates. The control strain ATCC25922 was within these major subclades. Genetic diversity was observed among the isolates despite their shared origin, phylogroup, or genotype.

## Discussion

The rising antimicrobial resistance rates among Enterobacterales, particularly *E. coli*, have been increasing in the past decade,^6^ and the COVID-19 pandemic may have accelerated this process.^20^ This poses a significant challenge to public health and clinical practice. Our isolates exhibited high resistance to commonly prescribed antibiotics, including ampicillin, ceftazidime, ceftriaxone and ciprofloxacin, with increased rates compared to local reports prior the pandemic.^21^ These findings highlight the need to consider new treatment options for these microorganisms and underscores the importance of antimicrobial stewardship to reduce the spread of resistance.

The presence of ESBL-producing *E. coli* strains complicates treatment, as β-lactams and quinolones are key in empirical therapy for CA-UTIs, according to the Infectious Diseases Society of America (IDSA) guidelines.^7^ Our findings indicate that the analyzed strains harbor genetic resistance determinants for both β-lactams and quinolones, limiting treatment options. Specifically, we found CTX-M to be the most prevalent type of β-lactamase, consistent with global trends where CTX-M variants can overshadow other β-lactamases such as TEM and SHV.^4^ Interestingly, while the widely distributed variant CTX-M15 was absent in our strains, CTX-M2 was the most frequently detected, aligning with other regional studies[1,3] and contrasting previous reports from other areas in Mexico,^22^ Notably, 96.6 % (86/89) of the strains contained CTX-M type genes from two or more different groups, a phenomenon with limited prior reports.^1^^,^^6^ The presence of CTX-M genes (98.9 %) likely explains the observed resistance to cephalosporins, since these β-lactamases can hydrolyze cefotaxime, ceftriaxone and ceftazidime.^4^ Although co-occurrence of CTX-M with other ESBL genes in a strain is not uncommon,^6^ this co-occurrence remains notable.

The prevalence of ESBL-producing strains may vary between community and healthcare settings. Studies show that ESBL rates tend to be higher within healthcare settings.^2^ In Mexico, several studies have focused on healthcare settings across different regions.^5^^,^^10^, ^11^, ^12^ In contrast, there are fewer reports addressing both community and healthcare settings,^6^^,^^12^ as well as fewer studies from community settings.^1^ Further research is needed to compare prevalence rates between healthcare and community settings within our region, to understand antimicrobial resistance dynamics in both contexts and to improve infections management.

Regarding the PMQR genes, the *oqxAB* (a multidrug efflux pump), *qepA* (efflux pump) *qnrB* (encoding a protein that protects type II topoisomerase), and *aac(6′)-lb-cr* (modified acetyltransferase) genes,^23^ can confer low resistance to quinolones when present in a strain. The most prevalent PMQR in our study was *aac(6′)-lb-cr,* which is consistent with previous reports,^11^^,^^13^ and was present in over 90 % of the isolates from healthcare institutions^5^ as well as in community settings.^12^ Our isolates were recovered from a community setting, an environment where resistance may be underreported.

The *aac(6′)-lb-cr* gene is associated with resistance to quinolones and can increase the Minimal Inhibitory Concentration (MIC) for ciprofloxacin,^23^ Interestingly, although this gene encodes an Aminoglycoside Modifier Enzyme (AME), we observed a different susceptibility pattern for amikacin and gentamicin, both of which belong to the aminoglycoside class. Amikacin exhibited greater susceptibility than gentamicin, which may be more readily modified by the acetyltransferase enzyme due to structural differences.

PMQR genes can also coexist in isolates, as previously reported in Mexico.^5^^,^^10^^,^^12^^,^^13^ The co-occurrence of PMQR could enhance resistance to quinolones by harboring different resistance mechanism against them.

Notably, five strains tested negative for all the PMQR genes analyzed in this study. While we employed specific primers for the *qnrB* variant^24^ identified as the most prevalent allele in previous research, other *qnr* alleles have been also reported in Mexico.^11^ Additionally, resistance could be mediated by mechanisms not covered in this study, such as mutations in the quinolone resistance-determining region of type II topoisomerase genes^11^ or other genetic determinants not assessed here. Further analysis, including sequencing of the *gyrA* and *parC* genes is needed to elucidate the specific mechanism of quinolone resistance in these strains. Given that PMQR genes are commonly found in clinical isolates alongside these mutations,^11^ additional investigations are necessary to confirm this association in our isolates.

The coexistence of ESBL and PMQR genes in *E. coli* has been previously reported.^12^^,^^25^ In our study, 82.07 % of strains carried *aac(6′)-lb-cr* and *bla_CTX−__M_.,* a combination often found in plasmids,^25^ and in strains colonizing healthy individuals.^22^ This suggests the potential for horizontal gene transfer to commensal strains, leading to multi-drug-resistant strains that are difficult to treat empirically.

The prevalence of phylogenetic groups can vary significantly by region. In our study, most isolates (74.2 %) belonged to Group B2, which has been associated with extraintestinal infections and antimicrobial resistance to β-lactams and quinolones.^8^^,^^26^ This finding is consistent with reports from Mexico, where Group B2 is predominant among UTI isolates.^1^^,^^22^^,^^27^ In contrast, group A is typically associated with intestinal commensal strains.^1^ Our results show that group A was the second most prevalent phylogroup (15/89), consistent with previous reports in Mexico, where it was also commonly recovered from UTI patients,^27^ and from intestinal *E. coli* isolates from healthy donors in northern Mexico.^1^ The presence of intestinal strains in UTI may suggest the risk of infection from commensal strains.

Our analysis revealed that 66.3 % (59/89) of the isolates were recovered from female patients, reflecting the higher prevalence of UTIs among women due to anatomical al physiological factors.^27^ Furthermore, 65.1 % of the isolates were recovered from patients over 55-years of age, suggesting that avanced age may be a potential risk factor for CA-UTI, independent of sex. A notable degree of antimicrobial resistance was also observed among the urinary isolates. Specifically, resistance to Trimethoprim/Sulfamethoxazole was found in 60.7 % of isolates despite its common use in UTI treatment.^7^ In contrast, the relatively low resistance rates observed for fosfomycin and nitrofurantoin (4.54 % and 1.12 %, respectively) suggest these agents may serve as viable alternatives for treating UTIs, although their use is limited for uncomplicated UTIs. Given that first-line agents recommended in Mexican guidelines^28^ may no longer be effective in most cases, alternative treatment options should be considered for complicated UTIs.

Several antimicrobial resistance profiles were identified among the isolates. The shared resistance to penicillins combined with β-lactamase inhibitors, cephalosporins, and quinolones suggest the presence of multiple resistance mechanisms across different antimicrobial classes. All strains were susceptible to tested carbapenems (doripenem, ertapenem, imipenem, and meropenem) and ceftazidime/avibactam. These antimicrobials could be considered as alternatives for complicated UTIs given the low resistance rates observed in this study. However, their use should be closely monitored to prevent the emergence of carbapenem-resistant strains.

To effectively combat antimicrobial resistance, ongoing surveillance of drug resistance and associated genotypes is essential.^6^ Several molecular typing techniques, including Multilocus Sequence Typing (MLST), Pulsed-Field Gel Electrophoresis (PFGE), and ribotyping, have been employed in epidemiological surveillance studies. In Mexico, PFGE has been the predominant method used in previous reports,^11^, ^12^, ^13^^,^^22^ however, ERIC-PCR also offers a more cost-effective and a strong discriminatory power for assessing clonal relatedness. ERIC-PCR has also been used in recent reports in Mexico.^3^ Our results revealed substantial genetic distance among strains, even within samples collected in the same community. The evolutionary distance increased as branches diverged from their common origin, suggesting that even strains sharing a resistotype can exhibit significant genetic divergence. This finding may result from ERIC-PCR amplifying intergenic fragments rather than resistance genes, which could have been acquired through MGE. The observed diversity could be attributed to the varied origins of patients. Mexicali is a city located on the Mexico-U.S. border and a popular region for medical tourism, attracting a diverse patient population. This diversity may contribute to the variation in community isolates and facilitate the spread of strains to other regions. Understanding these dynamics may aid in public health efforts aimed at control drug-resistant infections.

One of the main limitations of this study was the lack of detailed clinical information from patients, which prevented the identification of specific risk factors associated with CA-UTI. This limitation restricted a more in-depth epidemiological analysis of the clinical and sociodemographic determinants that may influence the acquisition and dissemination of resistant strains. Therefore, it is recommended that future studies incorporate broader clinical and demographic data to better understand the factors that contribute to the emergence and spread of multidrug-resistant strains in community settings.

## Conclusion

This study provides valuable regional data on the challenge posed by *E. coli* in community-acquired infections, particularly concerning antimicrobial resistance. To our knowledge, this is the first study to characterize the genetic resistance in *E. coli* within our community*.* The high prevalence of ESBL and PMQR genes associated with multidrug resistance limits available therapeutic options, complicating the effective management of these infections. The diversity of resistant isolates suggests multiple sources of these strains within the community, highlighting the need for enhanced antimicrobial resistance surveillance.

Given the observed resistance rates, exploring alternative antimicrobial classes and reassessing empirical treatment strategies are recommended. Understanding these patterns could help identify effective local therapeutic options and improve infection control strategies.

## Ethical approval

The study was performed following the guidelines of the General Health Law regulating human subject research, and following the principles of ethics established by the Declaration of Helsinki (1964) developed by the World Medical Association.

## Funding

This research did not receive any specific grant from funding agencies in the public, commercial, or not-for-profit sectors.

## Conflicts of interest

The authors declare no conflicts of interest.

## Appendix Supplementary materials

Image, application 1
