# Supplementary material for: Coexistence of PMQR and ESBL genes among clinical Escherichia coli isolates from community-acquired UTI in Mexicali, on the US-Mexico border
Source: Braz J Infect Dis. 2025 May 23;29(4):104554. doi: 10.1016/j.bjid.2025.104554 (PMC12153382; doi:10.1016/j.bjid.2025.104554)
Supplement: Supplementary file 1 [file mmc1.docx]

**BJID-D-24-00338_ Supplementary Material**

**Supplementary Table 1** Primer sequence and PCR amplification products used in this study.

| **Target** | | **Primer sequences (5’ to 3’)** | **Annealing temp. (°C)** | **Fragment size (bp)** | **Reference** |
| --- | --- | --- | --- | --- | --- |
| TEM | Forward | CAACATTTTCGTGTCGCCC | 55 | 844 | Arlet G et al., 1991 |
|  | Reverse | GCTTAATCAGTGAGGCACC |  |  |  |
| SHV | Forward | TATTATCTCCCTGTTAGCCA | 58 | 783 | (Garza-González et al., 2021) |
|  | Reverse | CGCTCTGCTTTGTTATTC |  |  |  |
| CTX-M 1y 8 group | Forward | TGTGCAGYACCAGTAARGYKATG | 55 | 583 | (Garza-González et al., 2021) |
|  | Reverse | TARRTSACCAGAAYVAGCGGC |  |  |  |
| CTX-M 1 group | CTX-M13U | GGTTAAAAAATCACTGCGYC | 50 | 843 | Briñas et al., 2005 |
|  | CTX-M13D | TTGGTGACGATTTTAGCCGC |  |  |  |
| CTX-M 9 group | Forward | ATGGTGACAAAGAGAGTGCAA | 55 | 747 | (Garza-González et al., 2021) |
|  | Reverse | AATATCATTGGTGGTGCCGTAG |  |  |  |
| CTX-M 2 group | Forward | CGATGTGCAGTACCAGTAAGG | 55 | 540 | (Garza-González et al., 2021) |
|  | Reverse | CGATATCGTTGGTGGTGC |  |  |  |
| CTX-M 151 | Forward | GCGGCCATGATAGGTACG | 55 | 786 | (Garza-González et al., 2021) |
|  | Reverse | AAAGTAAGTCACAATAACCAGCG |  |  |  |
| qepA | Forward | GCAGGTCCAGCAGCGGGTAG | 60 | 617 | (Qin et al., 2017) |
|  | Reverse | CCA CTG CTT GAG CCC GTA G |  |  |  |
| qnrB | Forward | GAT CGT GAA AGC CAG AAA GG | 53 | 594 | (Qin et al., 2017) |
|  | Reverse | ACG ATG CCT GGT AGT TGT CC |  |  |  |
| oqxA | Forward | CTC GGC GCG ATG ATG CT | 45 | 392 | (El-Badawy et al., 2017) |
|  | Reverse | CCA CTC TTC ACG GGA GAC GA |  |  |  |
| oqxB | Forward | TTC TCC CCC GGC GGG AAG TAC | 64 | 594 | (El-Badawy et al., 2017) |
|  | Reverse | CTC GGC CAT TTT GGC GCG TA |  |  |  |
| aac(6’)-lb-cr | Forward | TTG CGA TGC TCT ATG AGT GGC TA | 54 | 482 | (El-Badawy et al., 2017) |
|  | Reverse | CTC GAA TGC CTG GCG TGT TT |  |  |  |
